# A Method to Provoke Obsessive Compulsive Symptoms for Basic Research and Clinical Interventions

**DOI:** 10.3389/fpsyt.2019.00814

**Published:** 2019-11-11

**Authors:** Aron Tendler, Elyssa Sisko, Noam Barnea-Ygael, Abraham Zangen, Eric A. Storch

**Affiliations:** ^1^Brainsway Ltd., Jerusalem, Israel; ^2^Advanced Mental Health Care, Inc., Palm Beach, FL, United States; ^3^Department of Life Sciences, Ben Gurion University, Beer Sheva, Israel; ^4^Psychiatry and Behavioral Sciences Electroencephalogram, Functional Magnetic Resonance Imaging, Baylor College of Medicine, Houston, TX, United States

**Keywords:** deep repetitive transcranial magnetic stimulation, obsessive compulsive disorder, provocation, cognitive paired associative stimulation, exposure and response prevention

## Abstract

The efficacy of deep repetitive transcranial magnetic stimulation (dTMS) for obsessive compulsive disorder (OCD) was recently confirmed in a Food and Drug Administration-regulated, multicenter, sham-controlled study. In this study, patients who failed pharmacotherapy underwent individually tailored provocations just prior to each stimulation session, in the attempt to activate the relevant circuitry and making it labile to change. The procedure that was developed reliably evoked moderate intensity symptoms, making it effective on the one hand and mild enough to allow the patient to continue with the dTMS session on the other. This methodology article describes in a detailed step wise fashion how to evaluate the patient’s specific symptoms and design the individualized provocations. Additionally, the article explains how to instruct relevant personnel to administer the provocations, gauge their efficacy, and overcome possible obstacles. This method, apart from its ongoing role in the clinical treatment of OCD by dTMS, may be used for provocation of symptoms in basic studies [e.g., imaging with Electroencephalogram (EEG) or Functional magnetic resonance imaging fMRI] as well as other treatments.

## Introduction

In August of 2018, the Food and Drug Administration (FDA) cleared deep repetitive transcranial magnetic stimulation (dTMS) as a treatment for obsessive compulsive disorder (OCD) (1). The clearance was based on the positive results of a pilot (2) and subsequent multicenter trial (3) regarding both safety and efficacy. Both studies used the H7/HAC coil, placed over the medial frontal cortex (mPFC) and anterior cingulate cortex (ACC), and administered treatment sessions 5 days per week over 5–6 weeks. Patients had moderate to severe OCD despite prior pharmacological and/or psychosocial treatments, and the dTMS treatment protocol included individualized OCD symptom provocation immediately prior to each stimulation session. For each patient, this protocol was designed to achieve a moderate self-reported level of distress.

Provocations prior to dTMS, an approach called cognitive paired associative stimulation (4), has demonstrated better outcomes for the treatment of depression (5), smoking cessation (6), and post-traumatic stress disorder (PTSD) (7). This phenomenon can be explained, at least in part, by evidence suggesting that retrieval of items stored in long-term memory (e.g., by provocation) make it prone to change (e.g., by stimulation) (2, 8, 9). The authors of the depression study found that while emotional memories failed to further enhance the antidepressant effect of dTMS, negative emotional memories disrupted it (5). The authors of the smoking study suggested that rTMS interferes with the reactivated labile memory traces of cue-induced craving or inhibitory control circuitry (6), while the authors of the PTSD study suggested that stimulation enhances the consolidation of traumatic memories extinguishing the fear response (7). For a possible underling mechanism of provocation effect in dTMS-OCD studies, please see **Box 1**. For OCD, the effects of provocation were not controlled (i.e., all patients underwent provocation), but because of the excellent results demonstrated in published studies (2, 3, 10, 11) as well as patient acceptability, it continues to be used clinically.

Box 1 **Possible underlying mechanism of provocation in dTMS-OCD studies.**

Theoretically, activating obsessive-compulsive triggers may allow for the reconsolidation of fear or distress evoking memories, which are core to most forms of OCD, through dTMS. Experimental paradigms have focused on ways to inhibit or update fear memories in rodents and human adults (12–17). Once a memory trace is retrieved, it becomes labile for a period of time, before stabilizing again during the reconsolidation process (16, 19). Reconsolidation refers to the transfer of new information into long-term memories, resulting in revisions of the memory each time it is retrieved (20). Reconsolidation has been demonstrated in motor, declarative, and episodic memory (21, 22), although emotional memory is more resistant to change (23), necessitating a better understanding of processes that facilitate change in these pervasive fear memories. Disrupting the reconsolidation of a memory by electric stimulation has been done in both preclinical and clinical studies. For example, reconsolidation of conditioned taste aversion in the rat was impaired by localized intracranial electrical stimulation (24), while modification to existing human motor memory, reactivated by recall, were blocked by TMS-induced virtual lesion of the related cortical brain area (25). Disrupting the reconsolidation of a distressing memory by stimulating relevant OCD circuitry during this reconsolidation window (within 6 h of memory reactivation (22, 26) during which point memories are labile) has the potential to update the memory trace, enhancing and/or facilitating fear extinction. We acknowledge that an array of emotions characterize OCD symptoms, including fear, disgust and distress. The extent to which memory reconsolidation models translate to non-fear based psychopathology remains unclear, though initial data suggests potential applicability (27, ).

Support for updating fear memories during the reconsolidation window has been based on pharmacological studies (26, 29–31). In rats, infusion of propranolol, a β-adrenergic receptor antagonist, disrupted the reconsolidation of a reactivated fear memory (32). In humans, oral administration of propranolol prior to fear memory reactivation substantially weakened fear responses by disrupting the reconsolidation process (33). This procedure was specific to weakening emotional (fear) memories, leaving declarative memory intact (33). While propranolol and other pharmacological agents (e.g., anisomycin; MK-801) may serve to weaken fear memories or disrupt memory consolidation (26, 29), their use in applied clinical practice presents potential side effects and safety risks in humans. Beyond this, neuroimaging evidence in healthy adults shows diminished vmPFC involvement when introducing safety information when fear memories are labile (i.e., through symptom provocation) (34). This closely resembles the sgACC region that shows perturbations when appraising threat during extinction recall in anxious adults and youth (34).

Symptom provocation in OCD patients has a long history both in the study of the neuroanatomy underlying OCD and in the treatment of OCD using Exposure and Response Prevention (EXRP), which is the leading form of psychotherapy for OCD (35, 36). The primary distinction between symptom provocation and EXRP is the duration of the provocation as well as inclusion of varied cognitive and behavioral components (37). Symptom provocation is a brief procedure, inducing a few minutes of doubt while preventing the subject from performing the compulsion on its own; this is not a therapy. Brief symptom provocation for OCD was first used in the 1980’s to study regional cerebral bloodflow (38). Since then it has been used with positron emission tomography–computed tomography (PET-CT), single-photon emission computerized tomography (SPECT), and fMRI to study the functional neuroanatomy of OCD; results have been reviewed here (39–44). Since symptom provocation activates the OCD specific circuitry, a deep and broadly stimulating H7 coil will more selectively modulate that circuit. This is an example of Hebbian plasticity (45) induced by TMS, with the specificity of the circuit induced by the provocation (46, 47).

EXRP, first described in the 1960’s (48), is the first line treatment for OCD and the most essential component of cognitive behavioral therapy for OCD. It is therapist guided, and involves repeated extended exposure to distress-evoking triggers while abstaining from compulsions. While there is some variable in session length and duration of treatment, protocols typically involve 60–90 min sessions over 12–20 sessions (spaced once or twice weekly). The theoretical rationale underlying EXRP has been Emotion Processing Theory (49, 50), which relies on the reduction of within- and between-session distress as a core process underlying successful exposure therapy. Although both within- and between-session distress reduction—also known as habituation or extinction learning—likely plays a core role in EXRP, inhibitory learning deficits likely also play a role (50) in understanding the robust effects associated with EXRP.

This paper describes in detail how to design and administer the provocations, with detailed examples, under the notion that this method can be used both for basic research and for a variety of clinical treatments (51–53). While the underlying mechanisms of this approach remain largely theoretical with emerging empirical data, our primary goal was to articulate a clinical approach that represents a core component of dTMS treatment, which is becoming more commonly used given its efficacy (2, 3, 10, 11) and FDA clearance.

### Provocation Planning Guidelines

#### Educating the Patient

The patient should be educated about the nature of dTMS treatment, specific obsessions and compulsions, avoidance, and symptom provocation. Patients should be aware that prior to treatment their obsessive-compulsive symptoms will be provoked by a trained technician, and the purpose of provocations is for the treatment to be most effective. The provocation procedure aims to achieve a self-reported subjective distress score of 4–7 on a visual analog scale (VAS) ranging from 0 (no distress) to 10 (extreme distress), and patients should understand that greater or lesser levels of distress are not necessarily better and thus they should answer most accurately when asked about their symptoms. If they ever feel too distressed or that their obsessions are not being successfully provoked, they need to communicate this. The treating technician should reiterate these points at least weekly.

#### Educating Personnel

Relevant personal should be educated about OCD in general, the patients’ specific OCD, and the purpose of symptom provocation. Often, technicians feel uncomfortable provoking patients for various reasons, but typically they do not want to make the patient feel badly or hurt their feelings, do not understand the nature of the specific provocation, feel uncomfortable causing intentional distress for the patient, or discussing the topic of OCD (e.g., aggression, homophobia) with the patient. They must understand that eliciting obsessional distress is necessary and, although temporarily uncomfortable, it is helping the patient in the long run.

The technician should be instructed to not simply read out loud the provocations, but to understand and use them as a guide. When provoking the patient, it should be delivered as if the technician were having a conversation with the patient, sitting at eye level and making eye contact. It is helpful to engage in brief communication with the patient prior to initiating the provocation to gain information that could be used when starting the provocation. Brief small-talk frequently can be used as a conversation hook to make the provocation more natural, less forced and perhaps more potent than if the subject of their obsession is provoked directly.

The technician should be educated how to identify reassurance seeking behavior, and to not attempt to reduce the patient’s doubt (by giving reassurance) or allow them to perform any distress reducing behaviors or compulsions during the provocation process. Additionally, the technician should know how to look for verbal and body language cues for distress (e.g., looking away from trigger). Sometimes the patient subconsciously will tell the technician that something is not distressing, but in reality they are using avoidance. Lack of eye contact, clenching fists, muscle tension or rigidity, fidgeting, changing the topic of conversation, picking their skin and other hand activity, are all cues that infer the patient is experiencing more distress than they are aware or reporting. The technician should proceed with the provocation and when appropriate tell the patient what has been observed. It is also helpful to educate the technicians about tics, and how they increase with anxiety.

The technician should be flexible and supportive (although not reassuring) if the provocation takes longer than anticipated, and should continue from a different perspective such as the next provocation or pairing internal and external provocations. As the patient’s OCD improves over the course of treatment, it may increasingly challenging to elicit an appropriate level of distress upon provocation. It is possible that it may also take significantly longer if the patient is using significant avoidance and/or has poor insight.

#### Step-Wise Provocation Design

**Figure 1:**
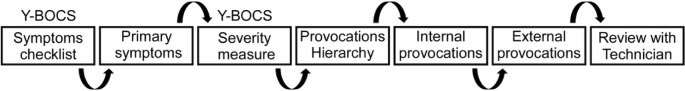
The seven-step process of provocation design.

Initially, a 90-min session to design the provocations is scheduled. The clinician rater meets with the patient to complete a seven-step process (**Figure 1**), beginning with the Yale-Brown Obsessive Compulsive Scale (YBOCS) symptom checklist (54). Detailed examples are provided at the end of the manuscript.

Step 1: Complete detailed YBOCS symptoms checklist to identify the current obsessions and compulsions. If the patient notes that symptoms are current and significant, ask for details and examples to understand the primary symptoms.

Step 2: Create a draft list of the primary symptoms. On the target symptoms form, ask the patient to list and quantify using the VAS their symptoms for obsessions, compulsions, and avoidance. Note the VAS scores of the patient next to each target symptom. If the patient gives a score that seems unreasonable, discuss with the patient and determine if the score provided should be modified. This list is later used to determine the most distressing or highest-ranking symptom.

Step 3: Complete the YBOCS severity scale assessing OCD symptom severity over the last week. This is done by the clinician using the target symptoms list and their knowledge of the patient’s functioning. It is not a patient rating scale and it should never be given to the patient to select.

Step 4: Create a hierarchy of symptoms from the draft list, such that the most prominent target symptoms are selected to create provocations to elicit moderate levels of distress. The provocations are separated into internal and external provocations. The internal provocations are questions related to thoughts, images, or impulses that usually trigger compulsive behavior, while external provocations typically involve requesting the patient to perform a task or involve the aid of an auxiliary tool.

Step 5: Develop internal provocations based on the hierarchy’s highest-ranking symptoms. These internal provocations are thought provoking questions related to the primary obsessions. The purpose is to engender a sense of doubt or create uncertainty with regard to the subject’s specific obsession, thereby increasing the subject’s level of distress. The internal provocations should be listed from least to most distressing in severity and the patient is prevented from performing compulsions or other safety behaviors until after the session is completed (although they are encouraged to do their best to not ritualize post-session). Examples of phrasing for internal provocations include: did you … this morning?; when was the last time you…?; is it possible that…?; how much does it bother you that…?; are you sure that….?; is there a chance that….?; and how can you be sure that….?

Step 6: Develop external provocations ranked from least to most distressing. External provocations are typically used after internal provocations are ineffective or in conjunction with them. External provocations usually use photos, auxiliary tools, or requesting the patient to complete part of a task. Examples of external provocations include: Write the distressing word on a piece of paper and show it to the patient; briefly show the subject a picture of …; place … near the subject in such a way that the subject is not sure whether or not he/she touched it; instruct the subject to touch…; have a mirror within eyesight but not where they can look at themselves; ask subject to start a list, email, text message and stop them after they start writing it; put an article about …. within view but do not allow them to see it; ask them to start a compulsion and interrupt them from completing it. Following the external provocation ask the subject: how much does it bother you that….?; is it possible that…?; and/or is there a chance that …? It is important that movement is relatively limited in the context of exposure to external provocations.

Step 7: The clinician should review with the technician the nature of the patient’s specific OCD. S/he should ensure it is understood that distress is necessary, and although uncomfortable temporarily, it is helping the patient in the long run. It is imperative to explain to the technician each patient’s specific case, the nature of the obsessions and compulsions, the reasoning, and any pitfalls or notes that may help them provoke the patient or triggers that should be avoided (e.g., too distressing). For example, a patient’s distress about dirt can be because the color of the dirt, the harm it may cause if it carries germs, the fact that it feels a certain way under their feet or hands, that the object is dirty or not clear, or something completely different. If the technician understands the reasoning behind the patient’s thought process, they can provoke the symptom using internal and external provocations as their guide. Technicians should be reminded to instruct patients not to attempt to reduce their doubt or perform any compulsions or avoidant behaviors during the provocation process. Technicians should not provide reassurance, rather keep the provocation going when the patient seeks reassurance. Clarify if they have questions and follow-up after the first treatment to see if there were issues, complications, or if clarification is necessary.

### Provocation Administration Guidelines

Provoke OCD symptoms utilizing the internal and external hierarchy provocation list. Use the questions created as a guide, but do not read the list. It is preferable to not even have the paper in your hands. Look at the patient in the eyes and speak to them as if you were having a conversation. Often, it is helpful to ask them questions about their day, which may provide details or a topic that can be used for provocation. This will make the process seem more natural to the patient. The purpose of the provocation is to instill doubt and to evoke a level of distress between four and seven on the VAS. The goal is for the current distress level to be in the moderate or higher severity range, not too mild to be ineffective and not too severe that the patient will be unable to sit through the session.

Begin with the first internal provocation, which is the least distressing OCD symptom on the list. Utilize the same internal provocation probe for several attempts before moving on to the next internal provocation, which is a topic of greater distress. Often you will need to keep creating doubt around that probe by planting several seeds or avenues of doubt. If that is ineffective in getting the patient to a 4–7 VAS score, continue through the internal provocations and then move to the external. Sometimes, it is beneficial to combine an internal and external provocation. For example, if a patient has distress involving being on time then the technician should hide all visible clocks in the office and could request the patient’s watch and phone upon arrival. The technician can do various activities that may appear to be delaying the treatment progress and/or frequently check their own watch. Additionally, the technician can ask the patient about their schedule, if there is a possibility they will be late or miss a call about another appointment, etc. Pairing the internal and external may be helpful so the patient cannot automatically complete the compulsion, and thus relieve their distress.

#### Troubleshooting

Several issues come up frequently. When a patient has poor insight, agreeing to sustained provocation is often difficult. For example, if a patient is unable to recognize that avoiding public places or buying a new car to avoid contact with contaminants is excessive or unreasonable, it may be difficult to get them to agree to do the task they are avoiding. Coincidentally, many patients also cannot be absent of an obsession or compulsion for long. This can be to the technician’s advantage when provoking in certain situations.

It is also not beneficial when the patient immediately completes a compulsion. For example, if they have an obsession and immediately (even when unaware) complete a compulsion that reduces their distress. The patient needs to be reminded to avoid completing compulsions prior to or during the session.

The purpose of starting at the least distressing internal provocation is to not provoke a level of distress above seven. If the patient is distressed to a higher degree, several options are available. The first approach should be to give a little time for the patient to calm down by talking about different subject or stepping out of the room for a few minutes. If necessary, the patient can be “talked down” but should not be given complete reassurance or allowed to complete a compulsion.

As OCD symptoms change, new provocations need to be created. That is, as stimuli become less distressing, developing new stimuli that evoke progressively more distress should be created. Additionally, as symptoms improve, provoking to the appropriate distress level becomes increasingly challenging. During this time, using internal and external provocations simultaneously is beneficial. Provocations that shift to consistently inducing no distress should be bypassed.

Patients may intentionally or unintentionally give inaccurate distress levels for various reasons, including poor insight, not understanding the purpose of the provocations, avoidance, difficulty quantifying the distress, and difficulty separating obsessional distress if OCD is extreme and constant. It is imperative to always keep open communication between the physician, technicians, and rater, to ensure that provocations are effective and that the patient is receiving the optimal treatment.

### Examples for Individualized Provocation

**Ethics:** The following case examples are from a multi-center randomized study, regulated by the FDA and Sterling Review Board in accordance with the declaration of Helsinki. All of the participants signed written informed consent to participate in the study and have their data published. Some details were intentionally written in a vague and more general fashion both to protect anonymity and because they are characteristic of many OCD patients.

### Patient A

Patient A is a 45-year-old Caucasian woman who works in the medical field. The OCD onset occurred at 7 years old; however, she first sought psychiatric help at age 28. Her current medications included Alprazolam 0.25 mg BID, Zolpidem 5 mg, and Paroxetine 60 mg. Previous medication history includes Paroxetine 30 mg for 3 years, Paroxetine 60 mg for 4 years, and sertraline 100 mg for 3 years. She had two courses of non-CBT forms of psychotherapy. Her OCD has impaired her social life and work significantly. Although she was presently employed, she was missing work often and had to switch positions due to her OCD symptoms.

**Table:** 

YBOCS SYMPTOM CHECKLIST:		
Current	Obsessions	Notes
__X__	Concern might harm others because not careful enough	
__X__	Concern may appear foolish in social situation	
P*__X__	Concern will be responsible for something terrible happening	fire, burglary**
**P**__X__	Other: Concern about having more anxiety	
	**Compulsions**	
**P**__X__	Checking locks, stove, appliances, etc.	*checking nothing is plugged in; checking tools or appliances are not hot*
**P**__X__	Checking that did not make a mistake	*mostly at work*
__X__	Need to tell, ask, or confess	*boss and spouse*
__X__	Other: need to drive self	
	**Avoidances**	
**P_**_X:	Avoids doing things, going places, or being with someone because of worries	

*P – primary symptom. ** Italicized words in parenthesis are examples of the type of notes the interviewer may write to remind themselves of details of the obsession or compulsion.

#### Symptom Hierarchy

Obsession 1: Concerns about disappointing or upsetting someone. (7)
Obsession 2: Concern they will make a mistake that will cause harm. (8)
Obsession 3: Concern about having anxiety. (10)
Compulsion 1: Check everything is unplugged and not hot. (5)
Compulsion 2: Need to drive herself. (7)
Compulsion 3: Need to confess. (7)
Avoidance 1: Avoids driving with other people. (7)
Avoidance 2: Avoids group social events. (6)

#### Internal Provocation:

1. Did you check everything was unplugged and/or turned off before you left the house? Is it possible you left something plugged in? How can you be sure nothing was warm?
2. Is there a chance you won’t be able to drive yourself later? Is it possible your battery may die or something happens to your car? How can you be sure?
3. Is it possible you upset someone today? How can you be sure you did not disappoint anyone? Is there a chance you are mistaken?
4. Is it possible you made a mistake today that could be harmful? How can you be sure you did not write something incorrectly or miss something? Is there a chance you are wrong?
5. Is it possible your anxiety will get worse today? How can you be sure you will make it through the day?

#### External Provocation

1. Give patient article about ways to prevent fires in the home. Stop her from reading it after approximately 30 seconds.
2. Have patient plug in a water boiler device in another room and leave the room with the device turned on and plugged in.
3. Take the patient’s keys.
4. Have patient start a text to someone asking if he/she upset or disappointed them. Stop patient before pressing send.
5. Have patient write down everything they did this week that may have caused harm. Stop them after writing 1 or 2 things down.

### Patient B

Patient B was a 46-year-old Caucasian woman whose OCD has always impaired her ability to work and her social life. She has no friends and rarely left the house. Her paternal grandmother, father, and brother all suffered from OCD. Her OCD onset occurred at 17 years old, and she first sought psychiatric help at age 25 years. She previously failed paroxetine CR 37.5 mg for 1 year and Bupropion for 1 year and had one suicide attempt. Although she had many years of psychotherapy, none was CBT. Her current medications were fluoxetine 80 mg, clonazepam 1 mg, quetiapine XR 300 mg, clonidine 0.1 mg, and buprenophine 2 mg.

**Table:** 

YBOCS SYMPTOM CHECKLIST:		
Current	Obsessions	Notes
__X__	Concern might harm others because not careful enough	*dog*
**P**__X__	Concern with dirt or germs	
**P**__X__	Obsession with order	*room, magazines, cds, closet, bathroom, clothes*
	**Compulsions**	
**P_**_X:	Excessive or ritualized bathing, toothbrushing, grooming, or toilet routine	*showering/washing hair*
__X__	Excessive or ritualized handwashing	
P__X__	Ordering/arranging compulsions	
	**Avoidances**	
**P**__X__	Avoids doing things, going places or being with someone that trigger rituals	*stores, leaving house*

#### Symptom Hierarchy

Obsession 1: Worrying about getting germs or dirty from this. (9)
Obsession 2: Worrying about dog. Is my dog ok? (9)
Obsession 3: Are my things arranged/ordered correctly? (10)
Compulsion 1: Washing hands. (8)
Compulsion 2: Bathing. (8)
Compulsion 3: Organizing closet, bathrooms, drawers, CDs, magazines. (9)
Avoidance 1: Avoids bathrooms in public. (8)
Avoidance 2: Avoids group social events. (6)

#### Internal Provocation

1. When was the last time you organized your books, CDs, &/or magazines? Is it possible they are not in perfect order? Are you sure the magazines are separated by type and in chronological order? Is it possible a magazine could be in a different category?
2. Is it possible your drawers are not in order? Is it possible something is in the wrong drawer? Are you sure the items are organized by size? Is there a chance you missed something? How can you be positive?
3. When did you last use the bathroom? Is it possible, you missed a spot cleaning? Are you sure nothing is on your countertops? Is everything organized with the items you use most in front? Is it possible, someone went in after you?
4. When did you last organize your closet? Is it possible there is a dry cleaning hanger? Are you sure the jeans are hung on the hanger the exact some way and by color? Is it possible the clothes should be classified as a different color? Are you sure all of the current season’s clothes are in the front of the closet?
5. How is your dog? Is it possible something is wrong with her? Are you sure?

#### External Provocation

1. Place magazines on table next to patient.
2. Website on computer about organizing your closet.
3. Place bathroom cleaning supplies in room so that patient can see it.
4. Article about germs in public places on table next to patient.
5. Website about tricks to cleaning your bathroom.

### Patient C

Patient C was a single, 27-year-old, Hispanic man. OCD onset occurred at 11 years old, and he first sought psychiatric help at age 21. He previously tried paroxetine HCl 20 mg for 2 months, sertraline 25 mg for 1 month, and clonazepam 3 mg for 1 month. No family history of OCD or suicide. His current medications included sertraline 150 mg and clonazepam 1 mg TID. His OCD symptoms significantly affected his functioning at school; he was failing half of his classes. Additionally, although social, he was unable to make friendships or romantically progress beyond the initial few dates.

**Table:** 

YBOCS SYMPTOM CHECKLIST:		
Current	Obsessions	Notes
__X__	Fear of blurting out obscenities or insults	
**P**__X__	Fear of doing something else embarrassing	
**P**__X__	Concern with dirt or germs	
__X__	Forbidden or perverse sexual thoughts, images, or impulses	
_____	Content involves homosexuality	
**P**__X__	Fear of not saying just the right thing	*socially with peers especially with women*
__X__	Concern with illness or disease	*STD*
**P**__X__	Excessive concern with aspect of appearance	*attractive*
	**Compulsions**	
__X__	Excessive or ritualized bathing, tooth brushing, grooming, or toilet routine	
__X__	Excessive or ritualized handwashing	
**P**__X__	Need to ask, tell or confess	
**P__**__X__	Checking tied to somatic obsessions	*mirror*
__X__	Other: checking social media	*facebook, dating applications*
	**Avoidances**	
__X__	Avoids going places because of obsessions	*public restrooms*

#### Symptom Hierarchy

Obsession 1: Am I attractive or not? (7)
Obsession 2: Did I do or say something that could be misinterpreted? (9)
Obsession 3: Thinking about what other people think about me? (10)
Compulsion 1: Checking to make sure not touching the toilet seat. (7)
Compulsion 2: Checking for photos on social media. (8)
Compulsion 3: Looking in mirror or reflective surface. (10)
Avoidance 1: Avoids bathrooms in public. (8)
Avoidance 2: Avoids group social events. (6)

#### Internal Provocation

1. When did you last spend time with a group? Is it possible you did something that they think is odd, weird, gross, or dumb? How can you be certain?
2. Who was the last person you asked about your appearance? Is it possible they think you are unattractive?
3. Is it possible you were misinterpreted? How can you be sure you were clear?
4. When was the last time you thought about someone’s opinion of you? Is it possibly they think negatively of you? Are you sure?
5. How do you know others don’t talk about you? Are you sure what your friends tell you is what they really think? Is it possible, you are wrong?

#### External Provocation

1. Leave article on table about prevalence of STDs.
2. Give him a mirror for 10–15 seconds, and then ask if he thinks he is attractive.
3. Leave homepage for Facebook on computer in front of the patient.
4. Ask patient to write a text to a friend and stop him before sending it.
5. Take photo of him for social media/dating website and ask him to post it.

### Patient D

Patient D was a 34-year-old Caucasian male who was married with children and worked in a retail store. His OCD onset was at 13 years old, and he first sought psychiatric help at 24 years old. He previously tried non-exposure-based psychotherapy, fluvoxamine 150 mg for 3 months, duloxetine 60 mg for 4 months, and aripiprazole 5 mg for 3 months. He had no family history of OCD or suicide. His current medications included fluoxetine 80 mg, clonidine 0.1 mg, clonazepam 0.5 mg, and gabapentin 600 mg. His OCD symptoms moderately affected his functions at work as well as socially.

**Table:** 

YBOCS SYMPTOM CHECKLIST:		
Current	Obsessions	Notes
**P**__X__	Content involves homosexuality	*is he gay? Straight? Bi?*
__X__	Other: Obsessions about what he would have to do to be gay	
**P**__X__	Concern with illness or disease	*cancer, heart disease, knee, pain, etc*.
	**Compulsions**	
**P** __X__	Checking tied to somatic obsessions	*sexually and health*
**P** __X__	Mental rituals (other than checking/counting)	*tells self: loves wife*
__X__	Need to tell ask, or confess	
	**Avoidances**	
__X__	Avoids doing things, going places or being with someone that trigger rituals	

#### Symptom Hierarchy

Obsession 1: Health: Is something wrong, will I get a disease, will I need pain medications and never get off them, will I get cancer, etc.? (9)
Obsession 2: Gay: Do I like men more than just as friends? How would I act and dress if I were gay? Did I look at that guy and feel more than when I look at a girl? (8)
Compulsion 1: Health: Going to doctors, researching symptoms, researching disorders, looking at health forums, webmd, researching or getting tests and procedures to check if something is wrong. (8)
Compulsion 2: Gay: Checking if he looks at a guy then girl or vice versa he feels more for the girl. (8)
Compulsion 3: Mimics sounds he feels relate to gays. (8)
Avoidance 1: Avoids using internet. (4)

#### Internal Provocation

1. Are you gay? Are you sure? Did you see any gay men today? Did you think about what you would wear? What your voice would sound like? Are you sure it wouldn’t sound gay? Did you think how you would change to become more feminine? What if you found out you were gay? How do you know you are not? Are you sure?
2. Did you look at a man to see if you felt like he was more than your friend? Have you thought about the guy on the rollercoaster? How do you know you were just having a great time? Are you sure it’s not more than that? What about your friend at baseball? Do you have emotional feelings for him? Have you compared it to women? Are you sure you like them more? How do you know?
3. Do you have any doctor’s appointments? Are you sure the doctors diagnosed you correctly? How do you know? What if you didn’t tell them all your symptoms? What if they missed something? How do you know they shouldn’t run a test again? Have you checked them online? Are you positive you did not miss something?
4. What if the XRAYs or tests give you a disease? How do you know you won’t get cancer from the radiation? What if a procedure causes more harm? How do you know the right test or treatment option was chosen?
5. What if you injure yourself or get in a car accident? Do you think you will be in pain? What if you need pain pills? What if you need to use them for a long time? Do you think it will affect your relationships? Work? How do you know?

#### External Provocation

1. Play a high pitch voice.
2. Show WebMD on the computer in front of him.
3. Put a pile of printed test results in front on the table.
4. Picture of an attracticve guy in short presentation.
5. Newspaper stories about a subject/person who found out they were ill surprisingly.

### Limitations

The primary limitations of this methodology lie in the fact that OCD symptoms are so variable and so subjective. One of the goals of cognitive paired association/state dependent TMS/provocation before stimulation or provocation before imaging is to reduce the variability that is inherent in a patient sample due to the state of the brain being variable. Future studies should investigate the efficacy of active and sham dTMS for OCD in a specific OCD subtype, i.e. harm OCD, using positive provocations, neutral provocations, and harm provocations. Additionally, objective physiological measures of negative valence should be taken along with brain activation to assess for the efficacy of the provocation.

## Data Availability Statement

The datasets generated for this study will not be made publicly available. It outlines a method for how to provoke OCD symptoms in patients for research or clinical interventions; therefore, it does not have datasets.

## Ethics Statement

The multi-center study that utilized this protocol for provocation was carried out in accordance with the recommendations of Sterling IRB, with written informed consent from all subjects. All subjects gave written informed consent in accordance with the Declaration of Helsinki. The protocol was approved by Sterling IRB.

## Author Contributions

All of the authors contributed significantly to both the content and presentation of the manuscript.

## Conflict of Interest

AT has a financial interest in Advanced Mental Health Care, Inc., a clinical and research TMS center and in Brainsway, the manufacturer of the dTMS systems. AZ is a cofounder of and has a financial interest in Brainsway. ES is employed by Advanced Mental Health Care, Inc. EAS receives research funding from NIH, the Red Cross, Greater Houston Community Foundation, Rebuild Texas, and Texas Higher Education Coordinating Board. He receives book royalties from Elsevier, Wiley, American Psychological Association, Lawrence Erlbaum, Springer, and Jessica Kingsley. He consults from Levo Therapeutics.

The remaining authors declare that the research was conducted in the absence of any commercial or financial relationships that could be construed as a potential conflict of interest.
